# Biofabrication of Endothelialized, Intrinsically Vascularized 3D‐Printed Recombinant Spider Silk Scaffolds

**DOI:** 10.1002/adhm.202504883

**Published:** 2026-02-08

**Authors:** Claire M. Weinhold, Stefanie Heltmann‐Meyer, Xuen J. Ng, Thomas Scheibel, Tobias Fey, Harald Wajant, Carol Geppert, Andreas Arkudas, Dominik Steiner, Raymund E. Horch

**Affiliations:** ^1^ Department of Plastic and Hand Surgery University Hospital of Erlangen Friedrich‐Alexander‐Universität Erlangen‐Nürnberg (FAU) Erlangen Germany; ^2^ University Center for Orthopedics Trauma and Plastic Surgery University Hospital Carl Gustav Carus Dresden Germany; ^3^ Department of Medicine 1 University Hospital Erlangen Friedrich‐Alexander‐Universität Erlangen‐Nürnberg Erlangen Germany; ^4^ Department of Biomaterials University of Bayreuth Bayreuth Germany; ^5^ Department of Materials Science and Engineering Friedrich‐Alexander‐University Erlangen‐Nürnberg (FAU) Erlangen Germany; ^6^ Division of Molecular Internal Medicine Department of Internal Medicine II University Hospital Würzburg Würzburg Germany; ^7^ Institute of Pathology University Hospital of Erlangen Friedrich‐Alexander‐Universität Erlangen‐Nürnberg (FAU) Erlangen Germany; ^8^ Comprehensive Cancer Center Erlangen‐EMN (CCC ER‐EMN) and Bavarian Cancer Research Center (BZKF) University Hospital Erlangen FAU Erlangen‐Nürnberg Erlangen Germany; ^9^ Department of Hand, Plastic, Reconstructive, and Burn Surgery BG Trauma Clinic University of Tübingen Tübingen Germany

**Keywords:** biofabrication, bioprinting, hydrogel, spider silk, T17b EPCs, tissue engineering

## Abstract

Tissue engineering aims to create functional tissues for regenerative medicine, where scaffold design and vascular integration remain key challenges. Therefore, the capability to promote vascularization, long‐term stability and biocompatibility are important requirements to a scaffold material. One goal is to optimize the cell‐to‐scaffold material interaction to support vascularization and de novo tissue formation. This study evaluates 3D‐printed and non‐printed recombinant spider silk protein eADF4(C16)‐RGD hydrogels in a rat arteriovenous (AV) loop model. The hydrogels were implanted subcutaneously using polytetrafluorethylene (PTFE) chambers, where the lower half contained an acellular 3D‐printed spider silk hydrogel, while the upper half either contained a manually extruded eADF4(C16)‐RGD hydrogel without cells (group A) or with T17b endothelial progenitor (EPCs) cells embedded (group B). Constructs were explanted after 2, 4, and 12 weeks. The 3D‐printed eADF4(C16)‐RGD scaffolds showed good biocompatibility and vascularization. Interestingly, the presence of T17b cells resulted in an increased biodegradation, with the 12 week constructs nearly completely dissolved. The cell‐laden constructs showed a significantly increased vascular density per construct area after 4 weeks compared to the cell‐free constructs. This study demonstrates that both the scaffold ultrastructure and the integration of T17b cells are effective strategies to enhance the functionality of biomaterials for tissue engineering.

## Graphical abstract

This study evaluates 3D‐printed recombinant spider silk hydrogel eADF4(C16)‐RGD in a rat AV loop model for tissue engineering. Constructs with T17b endothelial progenitor cells showed enhanced vascularization and biodegradation. Results highlight the importance of scaffold design and cellular integration in improving vascular density and overall biomaterial functionality for regenerative medicine.

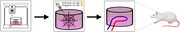

## 1 Introduction

Full or partial organ loss of function represents a serious impairment to a patient's health and quality of life to which currently there are only limited solutions. The restoration of form and function is one of the primary goals of regenerative medicine. Despite the considerable advances made in transplantation medicine there are still significant limitations in terms of the number of available donor organs, as well as challenges related to immune compatibility and organ rejection. The restoration of large‐volume tissue defects using autologous tissue transfer may also be constrained by various factors, including donor‐site morbidity, limited tissue availability, and/or the physical condition of the patient. The utilization of bioartificial tissues has the potential to enhance the accessibility of immunocompatible organs or tissues. Concurrently, this treatment approach could reduce morbidity and enhance the quality of life for affected patients. According to the definition of Langer and Vacanti, tissue engineering (TE) is an interdisciplinary field at the interface of biology and engineering that aims to develop a bioartificial substitute in order to restore or replace tissue [1]. An advancing subarea in this field is the development of active drug‐producing tissue containers, which enables the continuous delivery of therapeutics for the treatment of chronic diseases. The production of bioartificial tissue relies on the fulfillment of three distinct requirements that are directly linked to each other: the scaffold, its capacity to be vascularized, and the transplanted cells. The scaffold is the carrier matrix that houses the tissue‐forming or drug‐producing cells. Ideally, a scaffold should be biocompatible, biodegradable, and pro‐angiogenic, ensuring that the rate of biodegradation matches the rate of new tissue formation.

Hydrogels fulfil many of these criteria. Due to their high water content, they closely mimic the physical and biochemical properties of the natural extracellular matrix, providing a highly supportive microenvironment for the survival, proliferation, and functional integration of transplanted cells [2].

Recombinant protein‐based hydrogels have distinct advantages because their molecular composition and biofunctionality can be precisely controlled through chemical modifications and molecular engineering. A variety of different materials have been tested for TE applications, such as fibrin, resilin or recombinant spider silk protein [3, 4, 5, 6, 7].

Another example for materials in TE are resilin‐like polypeptides (RLPs). They can be crosslinked to form hydrogels with tunable properties. Modular RLP‐based hydrogels have been shown to support cell attachment and metabolic activity while exhibiting favorable mechanical behavior for mechanically sensitive tissues such as the human vocal folds [8, 9]. In vivo evaluation has also demonstrated their excellent tissue compatibility, with no detectable inflammatory reaction following implantation [10].

Building on these advances in recombinant protein hydrogels, RGD‐modified recombinant spider silk protein combines excellent biocompatibility with mechanical stability, controlled biodegradability, and had been shown previously to promote the formation of vascular networks in vivo [11, 12]. Due to these versatile properties, spider silk protein‐based hydrogels have been successfully applied in various biomedical contexts, such as in cardiac TE or bone engineering [13, 14].

In this study, we used the recombinant spider silk protein eADF4(C16)‐RGD, consisting of 16 repetitions of the C‐module, derived from the repetitive core segment of the native fibroin 4 protein from *A. diadematus* (the European garden spider) drag line silk, modified with an RGD‐containing peptide [15, 16]. Hydrogels made of eADF4(C16)‐RGD showed biocompatibility, high vascularization and slow biodegradation in vivo, suggesting this hydrogel to be a promising matrix for TE applications [11].

Besides the choice of scaffold, the supply of oxygen and nutrients via vascularization is a critical requirement for the survival of the transplanted cells. The microvascular endothelial cell network can provide oxygen and nutrients to the tissue‐engineered constructs, but diffusion is limited to 100–200 µm [17]. Therefore, it is necessary to integrate a new vascular network into the TE construct. Intrinsic vascularization by surgical‐induced angiogenesis through the formation of an arteriovenous fistula (AV loop) is a powerful method for tissue vascularization. In this regard, Erol and Sira have described the rat AV loop model forming an arteriovenous fistula between the saphenous artery and vein with a vein graft [18]. In the past, several biomaterials have been successfully vascularized, such as for bone or lymphatic TE [19, 20, 21].

To further improve vascularization or tissue formation, various cell types have been incorporated into scaffolds, such as mesenchymal stem cells, human umbilical vein endothelial cells, adipose‐derived stem cells, or cancer cells [7, 22, 23].

In this study, we implemented the murine endothelial progenitor cell line T17b as previously described [24]. It has been shown that T17b EPCs enhance blood vessel growth and exhibit low immunogenicity as they do not express major histocompatibility complex (MHC) molecules class 1 and therefore, are particularly suitable for xenotransplantation [25, 26]. With regard to a future drug‐producing tissue container, we used chemically transfected T17b cells, producing TNFR2‐Fc‐FLAG‐GpL, a therapeutic reporter protein consisting of the extracellular domain of tumor necrosis factor receptor‐2 (TNFR2) linked to the Fc domain of human IgG1 and the *Gaussia princeps* luciferase. The TNFR2‐Fc part of this reporter biologic correspond to Enbrel, which is used in scientific research and clinical practice as a tumor necrosis factor‐alpha (TNF‐*α*) inhibitor to treat inflammatory diseases such as rheumatoid arthritis, psoriasis and ankylosing spondylitis by blocking the TNF‐*α* cytokine. This plays a central role in the inflammatory response. Due to its GpL domain, TNFR2‐Fc‐FLAG‐GpL can be detected both in body fluids such as blood and urine and by virtue of its Flag tag also histologically in tissue sections [27].

In parallel, the advent of 3D printing technology has opened new avenues to create biomimetic constructs for improving vascularization by enabling precise modifications to the structure and porosity of scaffolds, thereby improving functional vessel density [28, 29]. Additionally, 3D printing has been shown to promote bone ingrowth, further demonstrating its potential in TE [30, 31].

This study evaluates the effects of a 3D‐printed eADF4(C16)‐RGD scaffold combined with T17b EPC encapsulation on vascularization as well as de novo tissue formation within an AV loop model for advanced TE applications. A combination of histological analysis and micro‐computed tomography (µCT) was used to assess these results.

## 2 Results

### 2.1 eADF4(C16)‐RGD Hydrogel Evaluation

**FIGURE 1:**
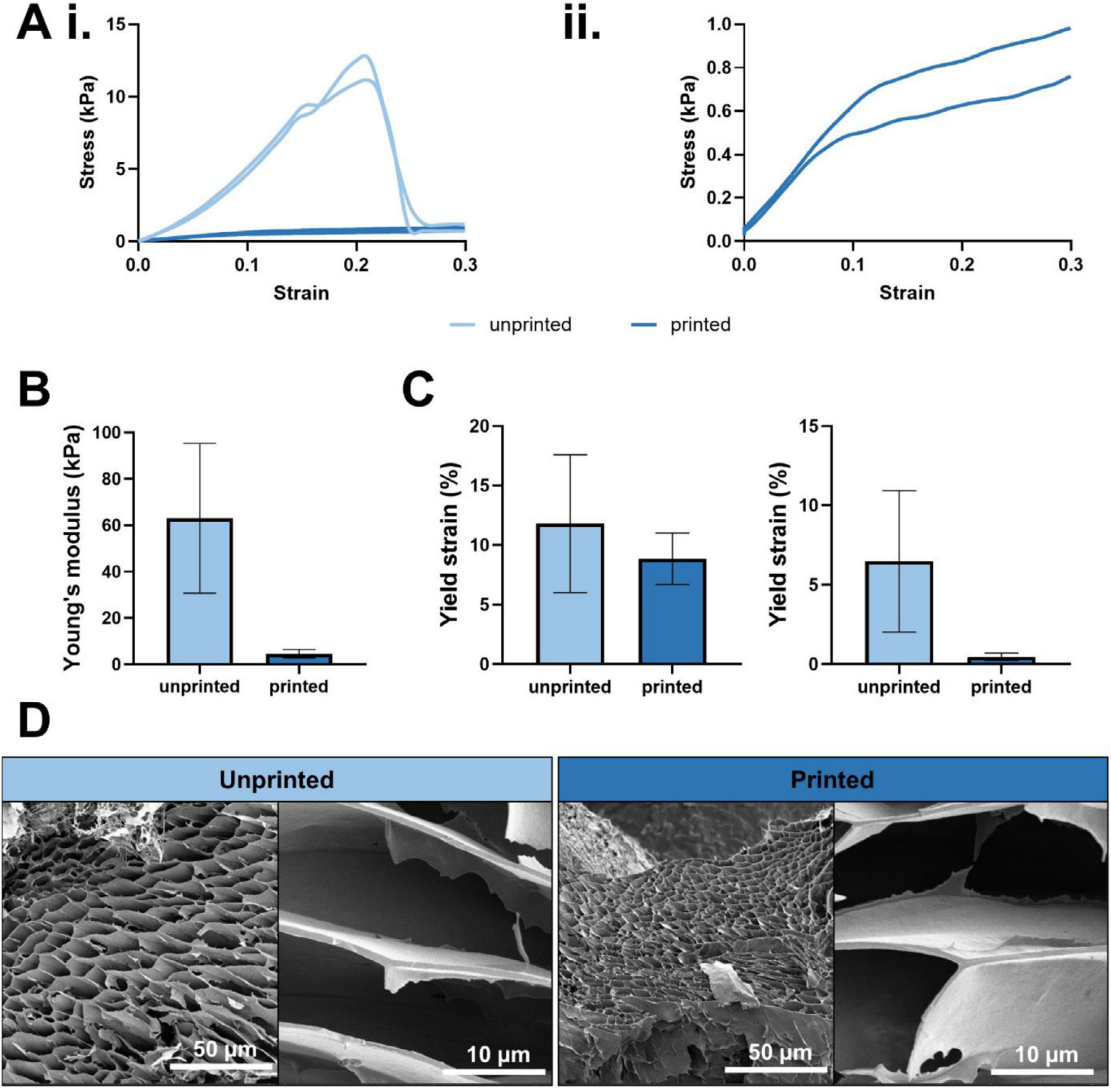
Mechanical properties and SEM images of printed and unprinted hydrogels. (Ai.) Representative stress strain curves of unprinted and printed hydrogel samples. (ii.) isolated stress‐strain curves of printed samples to emphasize plastic region. (B) Young's modulus and (C) strain and stress at the yield point. (n = 3). (D) SEM images of unprinted (left) and printed (right) hydrogel samples.

To understand the changes in bulk material mechanics post‐processing, we conducted compression experiments on printed cylindrical constructs (22 G, infill type: grid, 1 mm spacing) resembling the lower half within the implanted PTFE chambers and compared them to acellular unprinted hydrogel cylinders obtained from cut‐open cartridges. The stress‐strain curves of printed and unprinted hydrogel samples (Figure 1A) initially show a linear increase in stress proportional to the applied strain. Differences in bulk hydrogel structure resulting from the printing process, become apparent once the linear viscoelastic region (LVR) is exceeded. For unprinted hydrogels, a sharp drop in the stress‐strain curve is observed, followed by a second linear increase, and then later an ultimate drop in internal stress. In some cases, this was also an immediate observation and is due to material failure and hydrogel fracturing, with the extend of crack formation determining the magnitude of internal stress release (Figure S1). In contrast, for printed constructs the stress is not released in its entirety once exceeding the yield point (Figure 1Aii.). Instead, the stress gradually increases with a weaker slope. Once the initial bulk structure is broken, gels rapidly recover to an equilibrium for storage and loss modulus, but do not recover the entirety of the storable energy [27, 32]. We then compared the Young's modulus, the slope of the LVR (Figure 1B), and the stress and strain at the yield point (Figure 1C), the point after which plastic material deformation sets in. Unprinted gels had more than a ten‐fold larger elastic modulus compared to printed constructs (63.02 ± 26.38 kPa and 4.61 ± 1.48 kPa, respectively). Interestingly, the strain and stress at the yield point did not differ significantly between printed and unprinted samples. Scanning electron microscope (SEM) images show typical honey comb structure as reported previously [33]. Although no structural differences were evident, pores appeared smaller and walls thinner in printed constructs (Figure 1D).

### 2.2 Surgical Outcome

In the overall experimental setup, the lower half of the PTFE chamber was filled with the 3D‐printed eADF4(C16)‐RGD scaffold. Group A received an acellular, unprinted bioink of identical composition in the upper half of the chamber. In contrast, group B received unprinted bioink containing T17b cells in the upper chamber.

**FIGURE 2:**
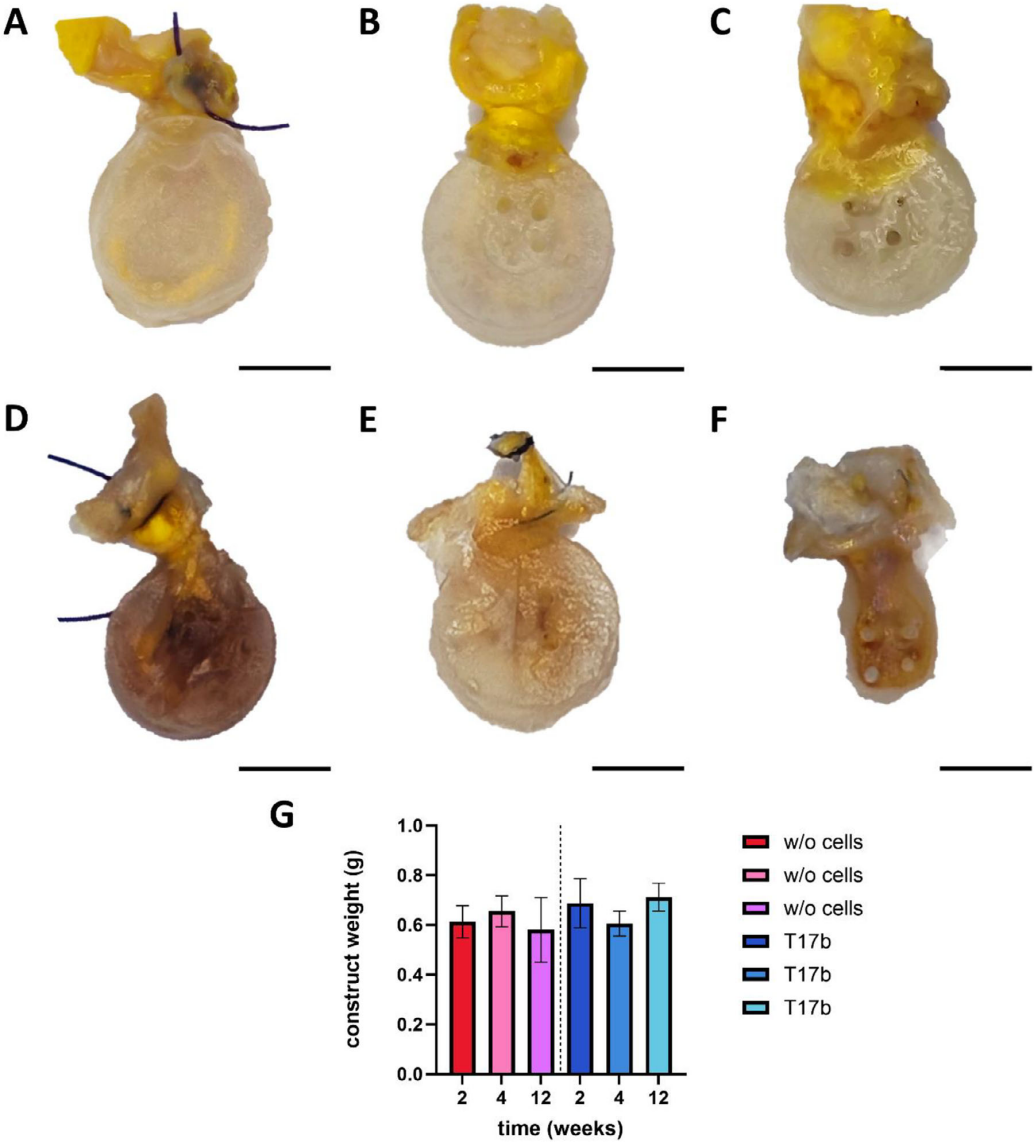
Macroscopic appearance and weight of explanted constructs. The vessels were successfully perfused with yellow Microfil. While the cell‐free constructs after 2 (A), 4 (B) and 12 (C) weeks and the 2‐week construct with T17b cells (D) appeared stable, the 4‐week constructs with T17b cells (E) appeared uneven and the cell‐laden 12‐week construct (F) showed almost no residual hydrogel. (G) No statistically significant difference in construct weight was detected between the groups. Scale bar = 5 mm (t‐test of same implantation intervals between the groups with and without cells n = 4–5).

One rat in group B displayed a wound healing disorder and was excluded from the analysis. No other surgical side effects were observed. To ensure that the rats were healthy, the body mass was monitored over the course of the study and did not lose any weight postoperatively. All PTFE chambers remained closed and undamaged. Apart from macroscopic images, the 12‐week explants were excluded from further evaluation. In the cell‐free group, the AV loops turned out to be not sufficiently perfused and in group B the constructs could not be removed from the chamber without damaging the remaining spider silk matrix. After excluding the 12‐week samples from each group, eight constructs per group remained. Six out of eight AV loops in group B (75%) and all eight AV loops in group A (100%) were patent. While appearing macroscopically stable after 2 and 4 weeks, the hydrogel matrix had almost completely degraded after 12 weeks of implantation in group B (Figure 2A–F). Construct weight was similar in both groups (Figure 2G).

### 2.3 Vascular Growth

**FIGURE 3:**
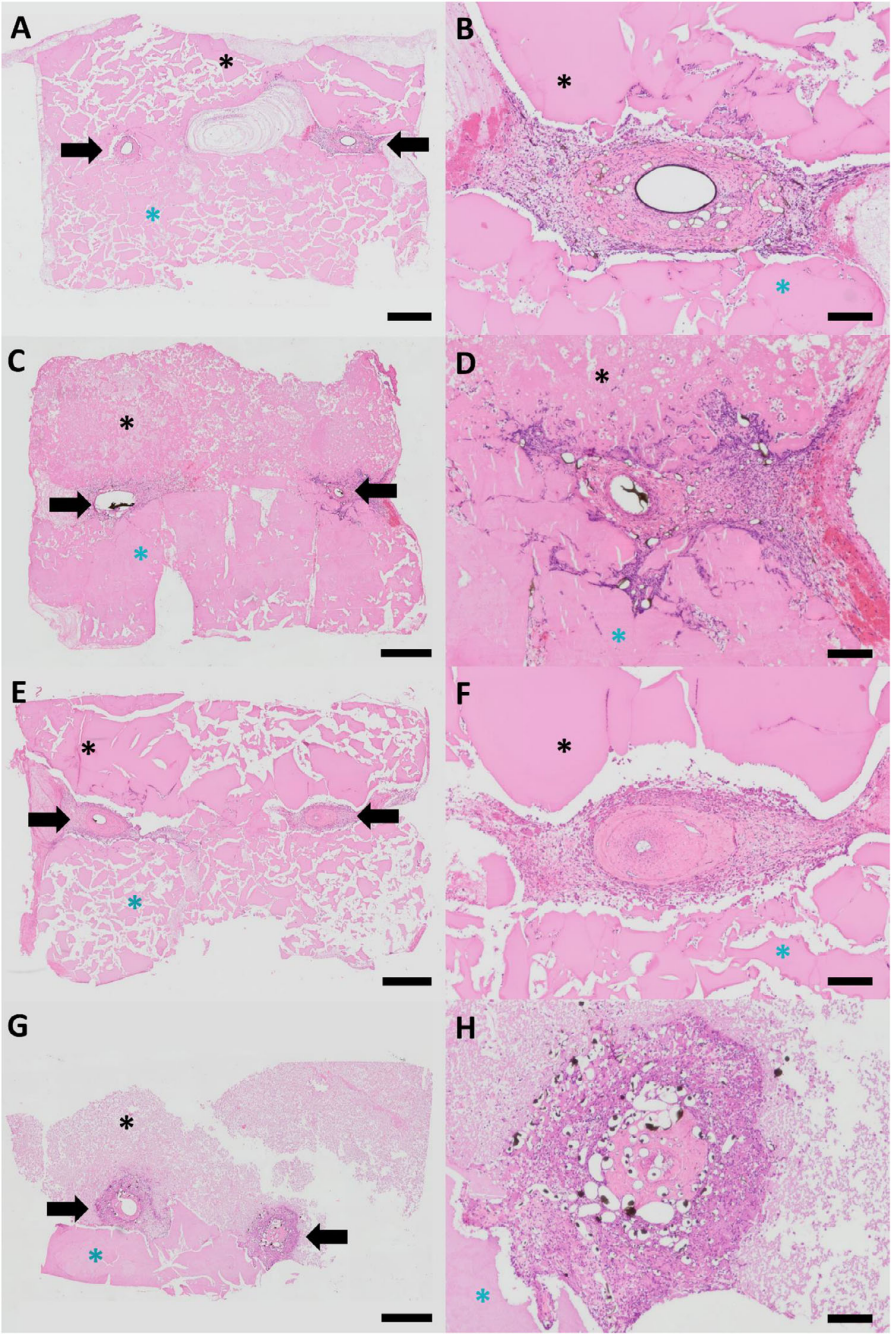
H&E staining after 2 and 4 weeks, displaying both overview and close‐up images in group A and B. After 2 weeks, the group without cells (A, B) and the group with T17b cells (C, D) showed no significant difference in construct size and the size of connective tissue. The 4‐week constructs containing T17b cells (G, H) were less structurally consistent than those without cells (E, F). Bold arrows point to the AV loop, the black asterisks indicate the cast spider silk and the blue asterisks mark the 3D‐printed spider silk hydrogel, black Microfil was only found inside the vessel lumen. Scale bar = 1 mm (A, C, E, G) and 200 µm (B, D, F, H).

Hematoxylin and Eosin (H&E) staining was used to quantify vessels, assess construct size, and measure the extent of newly formed tissue. After only 2 weeks, newly formed vessels and tissue were found around the AV loop. The lower half of the construct indicates the 3D‐printed spider silk scaffold, while the upper half exhibits the cast spider silk protein. The integrity of the two differently processed spider silks did not differ in group A. In contrast, the cast and cell‐laden spider silk protein in group B appears looser compared to the 3D printed part (Figure 3A–D).

After 4 weeks, a denser vascular network was observed in group B (Figure 3E,F). Moreover, the cast and cell‐laden upper half of group B appeared looser and the connective tissue has become more interwoven with the cast matrix containing T17b cells (Figure 3G,H).

**FIGURE 4:**
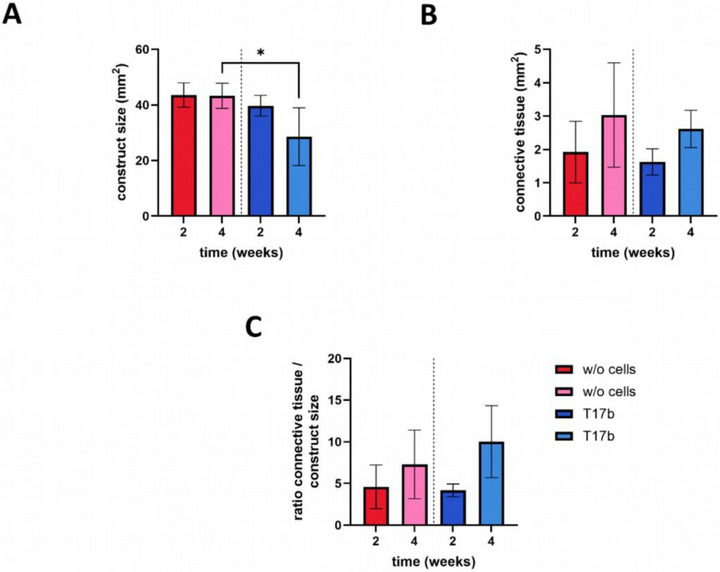
Construct and connective tissue size. The area of 4‐week specimens with T17b cells is significantly smaller than that of the 4‐week specimens without T17b cells (A). The experimental groups exhibit no statistically significant differences in connective tissue area (B) and connective tissue as a percentage of construct size (C). (t‐test of same implantation intervals between the groups with and without cells; * p ≤ 0.05; n = 3–4).

The cross‐sectional area (Figure 3), as an indicator of biodegradation, was quantified. Compared to the 4‐week constructs of group A, the 4‐week constructs of group B were significantly reduced in size (43.3 ± 7.5 vs 28.6 ± 10.4 mm^2^, p ≤ 0.05) (Figure 4A).

No significant differences were observed regarding the area of newly formed tissue at 2 and 4 weeks between both groups (group A: 2 weeks 1.92 ± 1.08 mm^2^; 4 weeks 3.03 ± 1.47 mm^2^; group B: 2 weeks 1.63 ± 0.39 mm^2^; 4 weeks 2.61 ± 0.56 mm^2^) (Figure 4B,C).

**FIGURE 5:**
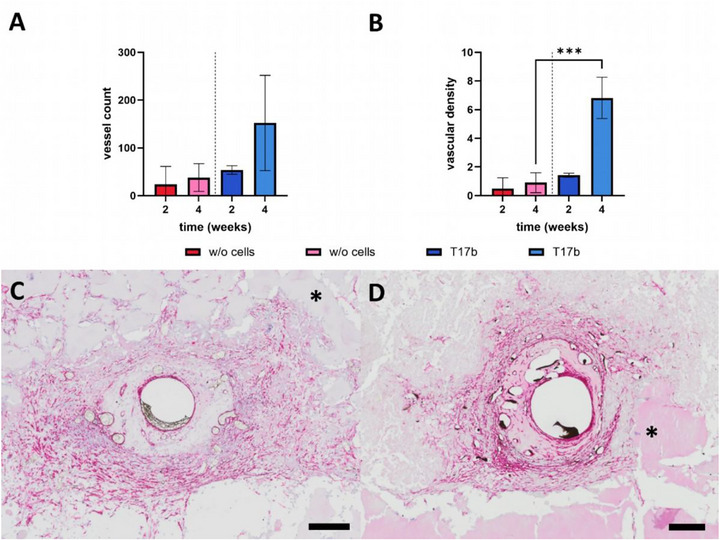
Quantification of the newly formed vessels and *α*‐SMA staining. There was no statistically significant difference in the number of vessels per histological cross section between the two groups. In relation to the construct area, significantly more vessels were detected in the T17b group after 4 weeks. *α*‐SMA staining of the 4‐week constructs without cells (C) and with T17b cells (D). The tunica media of the vessels was red‐stained, indicating the structural integrity. Black Microfil was only found inside the vessel lumen. Scale bar = 200 µm. (t‐test of same implantation intervals between the groups with and without cells; *** p ≤ 0.001; n = 3–4).

Newly formed vessels originating from the AV loop were observed in all groups (Figure 3). The 4‐week constructs of group B showed the highest number of vessel number per cross section of all constructs (152 ± 97) (Figure 5A). There was no significant increase in the number of vessels (group A: 2 weeks 24 ± 37; 4 weeks 38 ± 29; group B: 2 weeks 54 ± 9; 4 weeks 152 ± 100), but regarding the vascular density within the four‐week groups (group A: 0.90 ± 0.69; group B: 6.82 ± 1.44; p ≤ 0.001) (Figure 5B). The newly formed vessels were further analyzed with *α*‐SMA staining. We were able to demonstrate that most vessels displayed a media layer containing smooth muscle cells. (Figure 5C,D).

**FIGURE 6:**
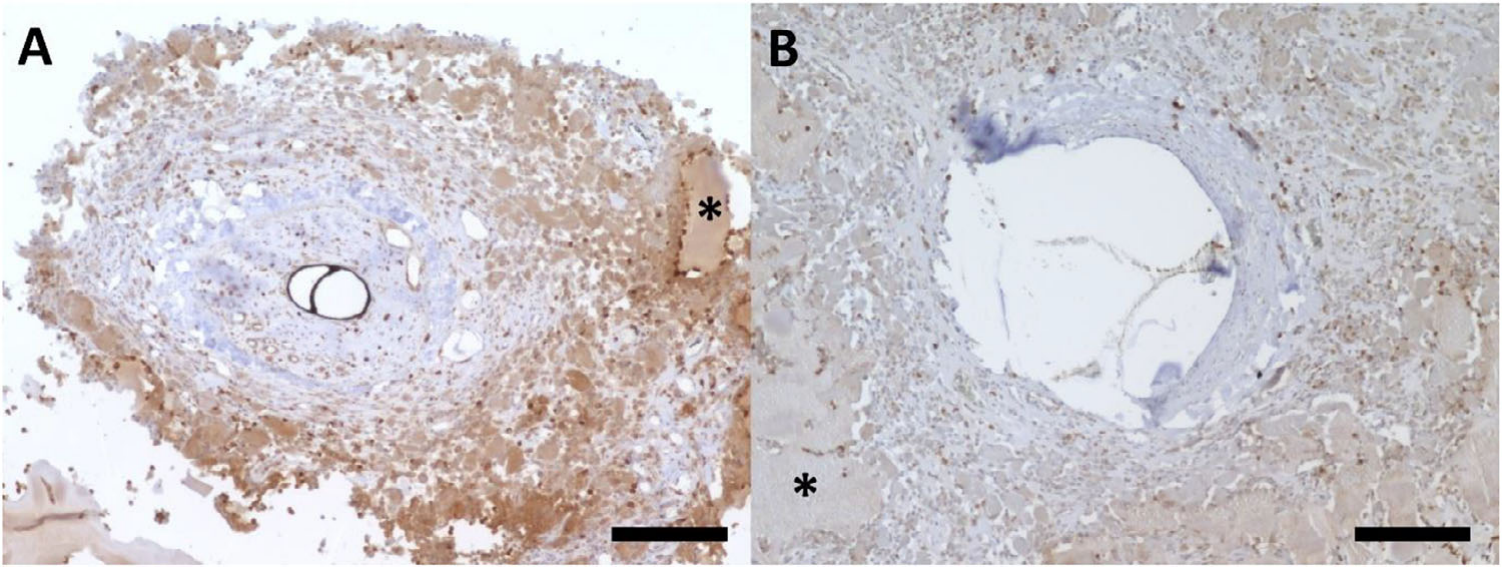
HIF‐1*α* staining. No apparent differences in HIF‐1*α* immunoreactivity were observed after 4 weeks without cells (A) and with T17b cells (B). Brown staining indicates HIF‐1*α*–positive nuclei. The asterisks indicate the spider silk hydrogel. Scale bar = 200 µm.

To examine oxygenation within the constructs, immunohistochemical staining for hypoxia‐inducible factor 1*α* (HIF‐1*α*) was conducted. It revealed no noticeable differences between acellular and cell‐laden constructs after 4 weeks. Both groups exhibited moderate, evenly distributed HIF‐1*α* expression in the connective tissue (Figure 6).

### 2.4 Immune Response and Matrix Degeneration

**FIGURE 7:**
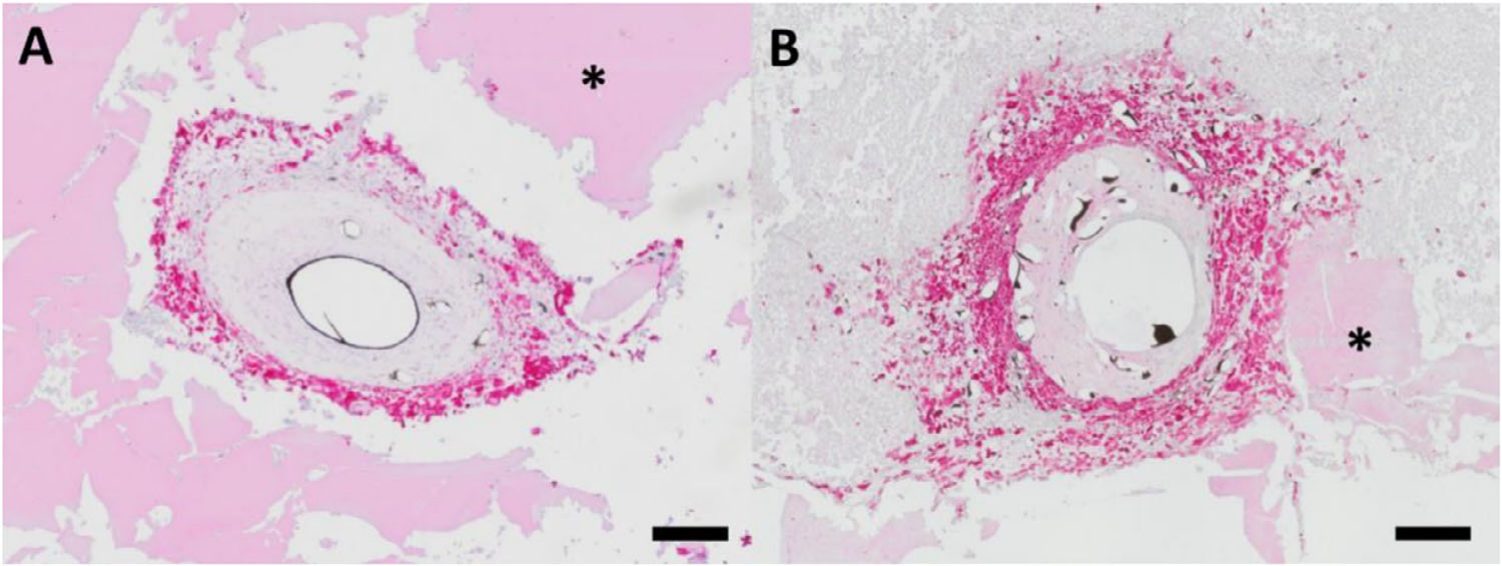
Macrophage staining. CD68 staining visualized red‐stained macrophages around the AV loop after 4 weeks without cells (A) and with T17b cells (B). No multinucleated giant cells were found. The asterisks indicate the spider silk hydrogel. Scale bar = 200 µm.

To further assess the immune response to the hydrogels, we used a CD68 staining to analyze the presence of CD68‐positive macrophages. While macrophages were found in the newly formed tissue in both experimental groups (Figure 7), no multinucleated giant cells were detected.

**FIGURE 8:**
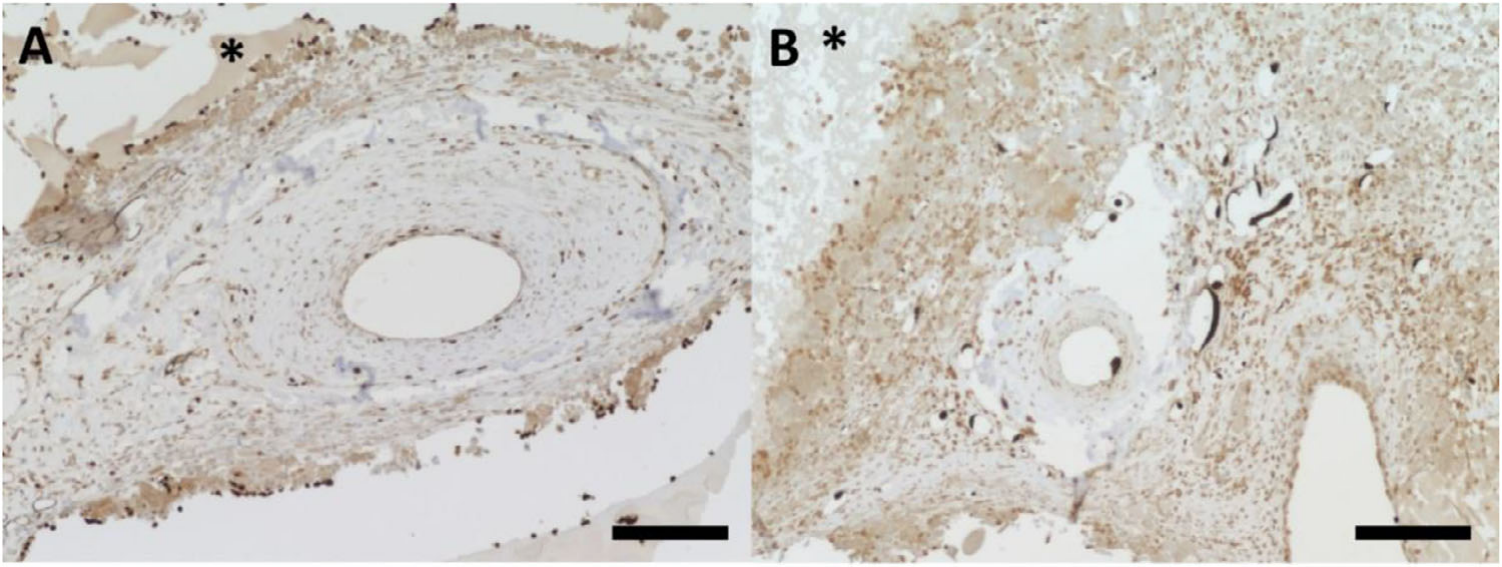
MMP‐3 staining. Brown staining indicates MMP‐3 positive areas in the 4‐week constructs without cells (A) and with T17b cells (B). In the group without cells (A), MMP‐3 staining is primarily observed at the interface between connective tissue and the spider silk hydrogel. With T17b cells (B), MMP‐3 signal is also present throughout the connective tissue. Black Microfil is visible inside the vessel lumen. The asterisks indicate the spider silk hydrogel. Scale bar = 200 µm.

As a marker for matrix degradation, matrix metallopeptidase‐3 (MMP‐3) was detected in both experimental groups after 4 weeks (Figure 8). In the constructs without cells (Group A), MMP‐3 expression was primarily observed at the interface between the connective tissue and the spider silk protein hydrogel. In contrast, constructs containing T17b cells (Group B) also exhibited MMP‐3 expression throughout the connective tissue. After 2 weeks, there was barely any MMP‐3 expression in either group (not shown).

### 2.5 µCT Analysis of the Vascular Network

**FIGURE 9:**
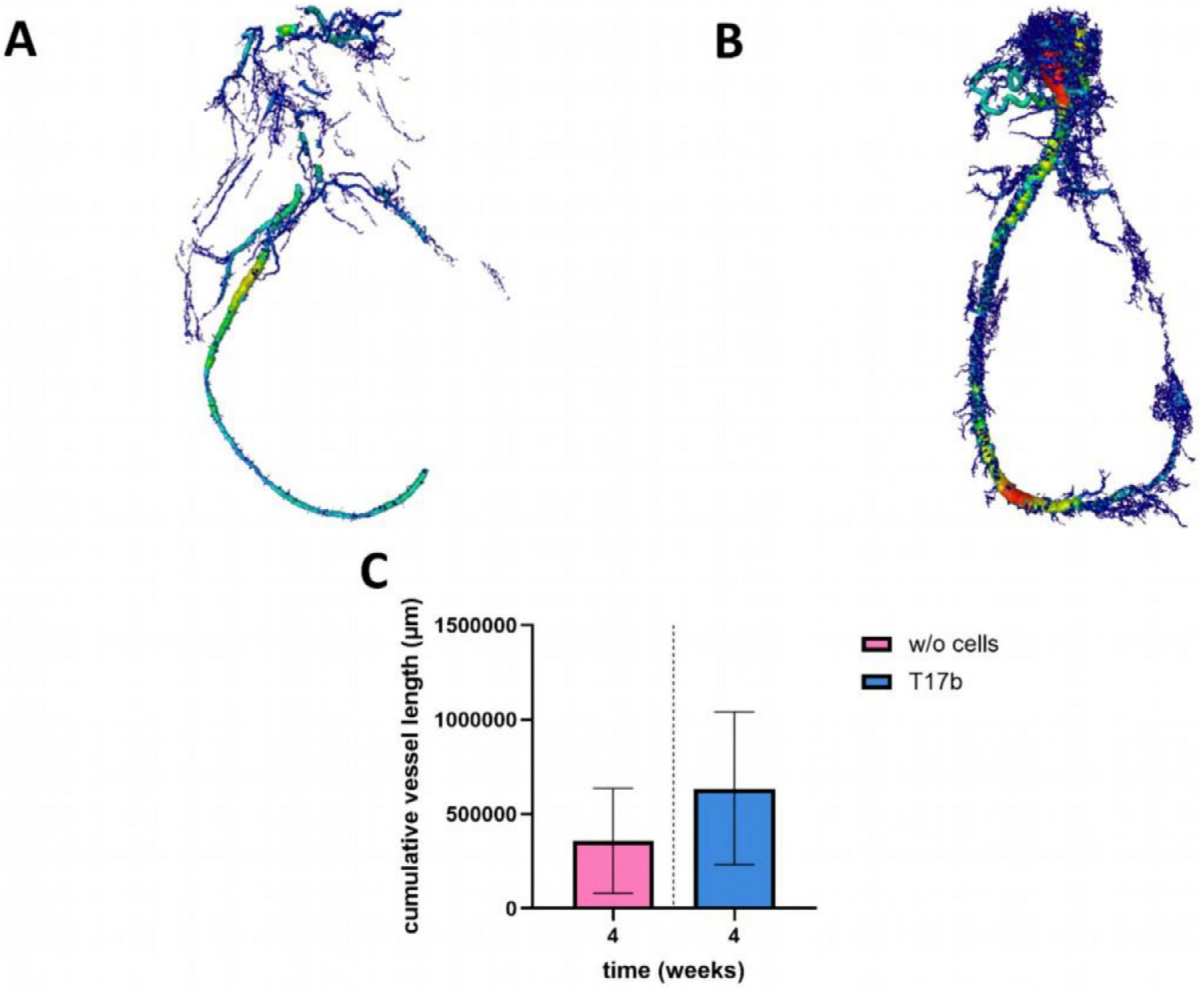
µCT analysis. µCT scan after 4 weeks of implantation without cells (A) and with T17b cells (B). Colors indicating vessel thickness. There is no significant difference in the calculated cumulative vessel length (C). (t‐test of same implantation intervals between the groups with and without cells; n = 3).

For 3D analysis of the vessel network, µCT was performed. The 12‐week specimen could not be evaluated because the AV loops in group A were not fully perfused with contrast agent and the samples in group B had been dissolved and were unstable. Sprouted vessels originating from the AV loop were detectable after 2 weeks of implantation. An increased cumulative vessel length of the 4‐week constructs of group B compared to the 4‐week constructs of group A was observed (636.1 ± 404.6 vs 358.3 ± 278.1 mm; n.s.) (Figure 9), consistent with the results of vessel number per cross‐section.

### 2.6 Protein Evaluation

**FIGURE 10:**
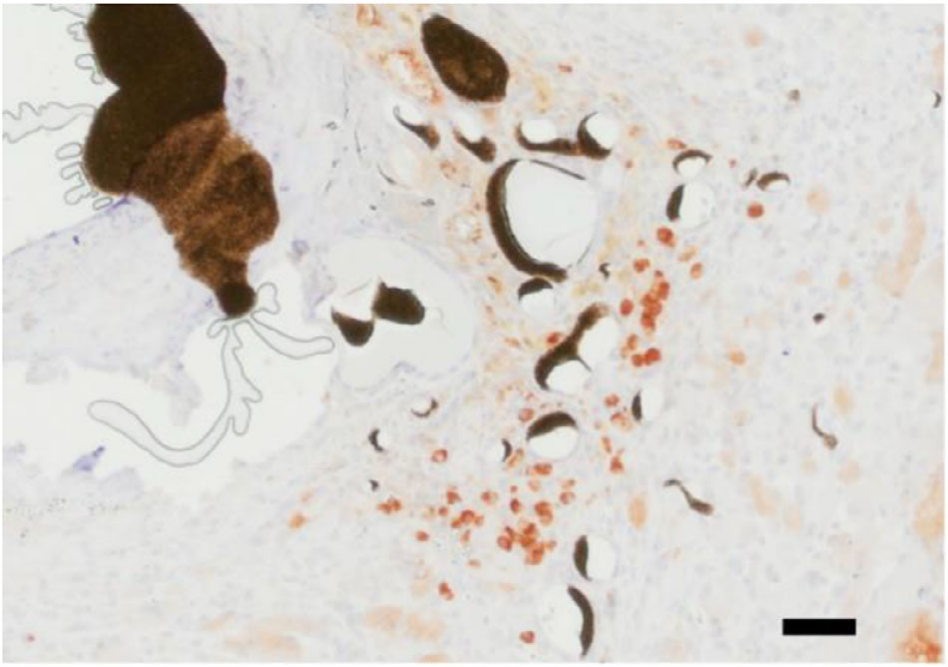
FLAG‐tag staining: The protein produced by the T17b cells was stained brown and was located within the newly formed connective tissue around the AV loop. Black Microfil was only found inside the vessel lumen. Scale bar = 50 µm.

No TNFR2‐Fc‐FLAG‐GpL fusion protein produced by the transfected T17b cells was detected in the system by blood and urine samples. To visualize the expression of the fusion protein inside of the construct, an anti‐FLAG‐tag staining was conducted. FLAG‐tag positive regions were found in the vascularized connective tissue around the AV loop after 4 weeks in group B (Figure 10).

## 3 Discussion

Tissue Engineering is of growing clinical importance in regenerative medicine to replace, restore and regenerate damaged tissue. A functional vascular network is critical for the survival and integration of engineered tissues, especially in larger constructs.

In this context, the recombinant spider silk protein‐based hydrogel eADF4(C16)‐RGD has emerged as a promising scaffold, as it fulfills all the requirements of a suitable hydrogel for TE in vivo and in vitro [11, 27]. Previous studies have shown that it is not only biocompatible, but also supports new tissue growth, improves mechanical stability, and promotes vascularization [11]. A major goal of the present study was to investigate the combined effects of 3D‐printing and proangiogenic cell encapsulation on construct integration and vascularization, as well as its suitability as a drug‐producing tissue container in vivo.

Various techniques were combined for this study. For instance, the hydrogel utilized in the study was 3D printed, and then implanted with an additional layer of cast cell‐free or cell‐laden hydrogel.

We were able to show that although unprinted constructs withstood higher compressive forces compared to printed samples, under compression, both printed and unprinted hydrogel exhibited an initial linear stress–strain response. This is expected, since the recombinant spider silk proteins form hydrogels through a self‐assembly process whereby hydrophobic regions in the repetitive core sequence form antiparallel beta sheets [33]. Through lateral assembly, nanofibrils and ultimately a rigid hydrogel forms with properties well‐suited for 3D printing [32]. The sharp drop of the unprinted hydrogels in the stress‐strain curve aligns with the brittle characteristics of non‐covalently and physically crosslinked silk protein hydrogels evident from rheological analysis of eADF4(C16)‐RGD hydrogels published previously [27, 32]. In contrast, 3D printed constructs did not fracture when reaching the yield point. In summary, the mechanical data aligns well with the observation that printed constructs tend to flow under force, whereas unprinted gels tend to fracture when the yield point is exceeded. This can be explained by the lower density derived from the infill pattern applied to the printed constructs additionally to the partial recovery of the storable energy post‐processing.

It was evident that there were differences in behavior when comparing the presence and absence of cells. Furthermore, a divergence in texture was observed between printed and unprinted spider silk protein.

The present study corroborated the biocompatibility of the hydrogel, notably with or without encapsulated T17b cells, as substantiated by the absence of multinucleated giant cells and the absence of any deleterious effects in the rats. A more comprehensive assessment, including analysis of M1 and M2 macrophages, was already conducted in a previous work, using the same recombinant spider silk protein (eADF4(C16)‐RGD) [11]. It was shown that neither a pro‐ nor an anti‐inflammatory subtype predominated, consistent with a low immunogenic response. Furthermore, Bleiziffer et al. demonstrated through ED‐1 staining that the xenogeneic transplantation of murine T17b cells into Lewis rats did not result in increased infiltration of macrophages compared to acellular constructs [34]. This finding is consistent with the absence of MHC I surface molecules and highlights the immunocompatible nature of these cells.

While the tissue architecture between the printed bottom half and the cell‐free top half of the constructs appeared similar in histological analysis, the cell‐laden spider silk protein in group B appeared less compact than the 3D‐printed cell‐free bottom half. This effect is even more pronounced after 12 weeks. It is interesting to note that the majority of the cell‐laden constructs were also clearly degraded macroscopically after 12 weeks. As demonstrated in previous studies, the spider silk hydrogel exhibits stability within the AV loop for a period of four weeks [11]. This phenomenon was further substantiated by the present study's findings in Group A regarding the construct size (here: 43.3 ± 7.5 mm^2^ vs. 41.1 ± 3.84 mm^2^) [11]. This finding indicates that 3D printing does not compromise the stability of the material. Conversely, Group B exhibited a substantial decline in stability after a period of 4 weeks. Macroscopically, it was evident that the implanted matrix was highly unstable after 12 weeks, and it could not be extracted intact from the implantation chamber. Concurrently, MMP‐3 staining revealed ongoing matrix remodeling after 4 weeks. In contrast to the acellular ones, those constructs containing T17b cells exhibited strong MMP‐3 expression in the connective tissue, suggesting that cell presence enhances matrix turnover, reflecting dynamic interactions between the cells and the newly formed connective tissue. Although accelerated matrix degradation was observed in constructs containing T17b cells after 4 and 12 weeks, the underlying mechanisms were not investigated in this study. Enzymes such as matrix metalloproteinases and cathepsins, which are known to participate in extracellular matrix remodeling, may have contributed to this process. Previous studies have demonstrated that endothelial cells can upregulate MMP expression in response to elevated levels of reactive oxygen species, potentially promoting enhanced matrix degradation [35, 36]. Alternatively, matrix breakdown could result from physical or mechanically driven processes, as the presence of cells may influence the self‐assembly or crosslinking behavior of the spider silk protein, potentially reducing structural stability.

As demonstrated before, scaffolds with larger pores exhibited a higher density of functional vessels compared to those with small or medium pores [28]. In our study, 3D printing of the spider silk hydrogel allowed the generation of constructs with pores approximately 1 mm in width, thereby increasing the overall porosity. Despite this design improvement, the 3D‐printed constructs did not show improved vascularization compared to the unprinted constructs described by Steiner et al. [11]. It appears that a boundary is forming in the direction of the 3D‐printed silk strands, indicating that angiogenesis occurs predominantly within the area of the cast gel. A reason could be the different hydrogel texture in the upper part of the chamber due to the high cell density and thus associated with the hydrogel's altered properties in terms of density and stiffness. This phenomenon could be due to the structural remodeling of the matrix of the cells [37, 38]. Cells can degrade matrices in such a way that the remodeling creates more space and stimulates vascular growth in the resulting space.

These results are consistent with previous experiments in which encapsulation with mesenchymal stem cells (MSCs) resulted in increased biodegradation of the hydrogel [22]. In line with this, the cells have been demonstrated to exert an influence on the vascular density, which is already elevated within the groups after two weeks and significantly increased after four weeks.

However, when compared to the unprinted, cell‐laden spider silk constructs of Osterloh et al., the 3D‐printed constructs showed a higher number of vessels per histological cross section after 4 weeks (here: 152 ± 100 vs. 115 ± 77) [39]. Notably, this difference was not present after 2 weeks, suggesting that 3D printing may influence vascularization at a later time point. For the future, optimization of pore size remains a goal, as smaller pores may limit permeability, while larger pores may reduce stability. In addition to enhancing the pro‐angiogenic properties of the scaffold, specific cell types can be encapsulated. As first described by Asahara et al., endothelial progenitor cells are known to enhance vascular growth [25, 40, 41]. We were able to confirm that incorporation of T17b cells slightly enhanced vascularization as shown by the increased number of vessels and cumulative vessel length in cell‐laden constructs after 4 weeks compared to the cell‐free group. The significantly higher vessel density relative to the construct size can be explained by the higher stability of the newly formed connective tissue, where all the vessels are located, compared to the remaining spider silk hydrogel. This result suggests that T17b cells promote angiogenesis and improve vessel growth compared to the cell‐free constructs. Adjustments to the cell concentration could be considered to further optimize vascularization and construct stability.

Taking these aspects into account, it can be considered that angiogenesis can be directed in future studies. It can be said that implanted cells have a positive influence on the remodeling of a matrix and thus promote angiogenesis at an early stage, while 3D printing is a tool that can positively influence the stability of the matrix, but only promotes angiogenesis at a later stage. In addition, 3D printing offers the possibility of adapting the matrix to the application in terms of shape, pore size and their mechanical properties. A combination of both systems could therefore contribute to directed angiogenesis and also promote cell migration into the gel and contribute to the formation of new tissue, thereby finding a balance between sufficient angiogenic stimulation and scaffold integrity [42].

Another important aspect of this study was the evaluation of the construct as a therapeutic tissue container. Despite the inability to detect the fusion protein systemically, histological analyses revealed its presence in the vascularized region of the implant. This finding suggests that the cells were adequately supplied by the main vessel, thereby supporting the hypothesis that sufficient cell survival occurred within the construct. Consistently, the lack of difference in HIF‐1*α* expression suggests that oxygen diffusion within the constructs was sufficient to prevent significant hypoxia, indicating that the scaffold architecture effectively supports an adequate oxygen supply. The presence of only negligible levels of the TNFR2‐Fc‐FLAG‐GpL fusion protein can be attributed to the specific cell line that was utilized in the study. The T17b cell line has not been documented as a high‐yield producer of recombinant proteins in the scientific literature. However, T17b cells possess the distinct advantage of lacking MHC I surface molecules, which allows for xenogeneic implantation without immediate immune rejection [24]. This distinctive property renders them especially advantageous in xenograft transplantation models.

In contrast, other cell lines, such as HEK293, are well‐established in the literature for their high protein production capacity [43]. These human cells have been utilized in prior in vitro investigations, wherein the efficacy of spider silk as a suitable matrix for such applications was demonstrated, thereby facilitating the diffusion of the fusion protein [27]. However, the use of human cells necessitates their implantation into immunodeficient animal models, such as RNU rats, in order to circumvent immune‐mediated clearance.

Whilst these immunodeficient models permit the transplantation of human cells, they are associated with considerable limitations. It is most notable that they are not suitable as inflammatory disease model as assessment of therapeutic efficacy in vivo. Consequently, a trade‐off exists between optimizing protein production and maintaining the biological relevance of the model. In order to enhance protein production within the current framework, it is recommended that the model be modified by increasing the number of implanted T17b cells. This approach has the potential to result in elevated levels of protein expression. However, it is important to note that this adjustment may also have consequences for the properties of the spider silk matrix, as well as the distribution of implanted cells in the construct. As previously discussed, alterations to the matrix could lead to changes in the mechanical and biological behavior of the silk. It is imperative that such alterations are given due consideration in forthcoming studies.

## 4 Conclusions

This study demonstrates successful vascularization of 3D‐printed eADF4(C16)‐RGD hydrogels combined with proangiogenic endothelial cells (T17b) in the rat AV loop model. We showed that the encapsulation of T17b was well tolerated in vivo with no observed severe immune response. Our results highlight that scaffold architecture and incorporation of T17b cells are valuable tools for modulating biomaterial properties, providing flexible strategies for adapting tissue‐engineered constructs.

## 5 Materials and Methods

### 5.1 T17b Cultivation

The cells from the murine endothelial progenitor cell line T17b (provided by the group of Prof. Stürzl, Division of Molecular and Experimental Surgery, Translational Research Center, Friedrich‐Alexander‐University Erlangen‐Nürnberg (FAU)) were seeded in culture flasks coated with 0.1% bovine skin gelatin type B (Sigma–Aldrich, Schnelldorf, Germany). The cultivation and differentiation protocol was initially described by Hatzopoulos et al. [24]. As culture medium, a high glucose Dulbecco's modified Eagle's medium (DMEM) GlutaMAX (Gibco/Life Technologies, Carlsbad, CA, USA) supplemented with 20% FCS Superior (standardized fetal bovine serum, Sigma–Aldrich), penicillin/streptomycin (100 U mL^−1^, 0.1 mg mL^−1^, Sigma–Aldrich), 1 mM nonessential amino acids (Gibco), 2 mM HEPES buffer pH 7.5 (Gibco), and 0.1 mM *β*‐mercaptoethanol (Gibco) was used. The EPCs differentiated into ECs by adding 1 µM all‐trans‐retinoic acid (ATRA, Sigma–Aldrich) and 0.5 mM dibutyryl cyclic adenosine monophosphate (cAMP, Sigma–Aldrich) to the cell culture medium 72 h before harvesting the cells.

The generation of the TNFR2‐Fc‐FLAG‐GpL producing T17b transfectants was essentially performed as described elsewhere for HEK293 cells [27]. In brief, T17b cells were transfected with the expression plasmid TNFR2(ed)‐Fc‐Flag‐GpL‐pCR3 using polyethylenimin and were then selected for stable transfection using G418. The amino acid sequence of TNFR2‐Fc‐Flag‐GpL is as follow:

MAPVAVWAALAVGLELWAAAHALPAQVAFTPYAPEPGSTCRLREYYDQTAQMCCSKCSPGQHAKVFCTKTSDTVCDSCEDSTYTQLWNWVPECLSCGSRCSSDQVETQACTREQNRICTCRPGWYCALSKQEGCRLCAPLRKCRPGFGVARPGTETSDVVCKPCAPGTFSNTTSSTDICRPHQICNVVAIPGNASMDAVCTSTSPTRSMAPGAVHLPQPVSTRSQHTQPTPEPSTAPSTSFLLPMGPSPPAEGSTGD**GS**KTHTCPPCPAPELLGGPSVFLFPPKPKDTLMISRTPEVTCVVVDVSHEDPEVKFNWYVDGVEVHNAKTKPREEQYNSTYRVVSVLTVLHQDWLNGKEYKCKVSNKALPAPIEKTISKAKGQPREPQVYTLPPSRDELTKNQVSLTCLVKGFYPSDIAVEWESNGQPENNYKTTPPVLDSDGSFFLYSKLTVDKSRWQQGNVFSCSVMHEALHNHYTQKSLSLSPGK**EF**DYKDDDDK**LE**KPTENNEDFNIVAVASNFATTDLDADRGKLPGKKLPLEVLKEMEANARKAGCTRGCLICLSHIKCTPKMKKFIPGRCHTYEGDKESAQGGIGEAIVDIPEIPGFKDLEPMEQFIAQVDLCVDCTTGCLKGLANVQCSDLLKKWLPQRCATFASKIQGQVDKIKGAGGD

The leader sequence is underlined, linker sequences are shown bold, the Flag tag is underlined with grey background, the Fc part has a grey background, and the TNFR2 and GpL parts are not highlighted.

### 5.2 Spider Silk Protein Preparation

The recombinant spider silk protein eADF4(C16)‐RGD comprises 16 repeats of a so‐called C‐module (amino acid sequence: GSSAAAAAAAA SGPGGYGPENQGPSGPGGYGPGGP) derived from the dragline silk of the European garden spider *A. diadematus* and was modified to contain the Arginine‐Glycine‐Aspartic acid (RGD) integrin binding‐motif using genetic engineering [15, 16]. The recombinant production and purification yielding the pure lyophilized recombinant spider silk protein are described elsewhere [15].

For preparing hydrogels, the lyophilized spider silk protein was first dissolved in 6 M guanidinium thiocyanate (Carl‐Roth, Germany) for 60 min at room temperature on an overhead shaker to ensure full solubilization. Subsequently the protein solution was filtered with a 0.2 µm syringe filter (Sartorius, Germany) and dialyzed against 10 mm Tris/HCl (pH 7.5) using dialysis membranes with a molecular weight cutoff of 6–8 kDa (Spectra‐Por, Fisher Scientific GmbH, Germany). To increase the protein concentration, the solution was dialysed against 25% (w/v) polyethyleneglycol (MW: 40000 Da, Carl‐Roth, Germany) which draws buffer from the solution through osmotic pressure. The protein solution was then collected, centrifuged and used to prepare a 35.3 mg mL^−1^ stock solution to which media was added to obtain a final media content of 15% (v/v).

For both groups one layer of the spider silk hydrogel was printed, therefore the prepared silk‐solution was transferred to pneumatic 3cc cartridges (Drifton, Denmark) for subsequent printing and left to gel through self‐assembly at 37°C and 5% CO_2_ in a cell culture incubator for about 16 h. For the upper half the silk‐solution was prepared with 15% (v/v) DMEM cell culture medium without (group A) or supplemented with 20 × 10^6^ differentiated T17b cells per ml (group B) and filled in syringes for casting later on.

### 5.3 3D‐Printing

Constructs were printed under a sterile workbench using a pneumatic direct extrusion print head on a regenHU Gen 1 bioplotter (regenHU, Switzerland) with a 22 G tapered tip (Drifton, Denmark) at a pressure of ∼0.84 bar and a print speed of 11.5 mm s^−1^. Five layers of a 10 mm diameter circle with a grid‐patterned infill of 1 mm spacing were directly extruded into PTFE chambers (10 × 10 × 6 mm). Chambers were then stored in culture media until implantation.

### 5.4 Mechanical Testing

Compression tests were performed on an Electroforce 3200 with a 2.45 N load cell (Bose, Bloomington, Minnesota, USA). Samples for printed constructs were printed into polystyrene cell culture dishes. Unprinted samples were taken from cut‐open cartridges as initial attempts to cast gels into PDMS molds failed due to drastic dehydration during the gelation phase (16 h at 37°C). Sample dimensions (diameter and height) were determined using images of samples mounted into the measuring set‐up by using the diameter of the stamp that was used to exert the force on the sample (12 mm) prior to testing. The speed applied for the indentation was 0.01 mm/s. Curves were smoothed using Origin applying the Lowess method set to 0.1 range. The Young's modulus was calculated from the slope of the LVR. Strain and stress at the yield point were determined from calculating the intersection of the slope of the LVR and the slope of the plastic region.

### 5.5 Scan Electron Microscopy

Scan electron microscopy images were acquired on a ApreoVS SEM device (ThermoFisher Scientific) using an ETD detector and applying a beam current of 25 pA and an acceleration voltage of 2.00 kV. Gel samples were dehydrated by snap freezing in liquid nitrogen and subsequently lyophilization. Dehydrated samples were then fractured and attached to sample carriers using Leit‐C conductive carbon ink (Plano GmbH) and coated with 1.3 nm platinum using an EM ACE600 (Leica).

### 5.6 AV Loop Surgery

**FIGURE 11:**
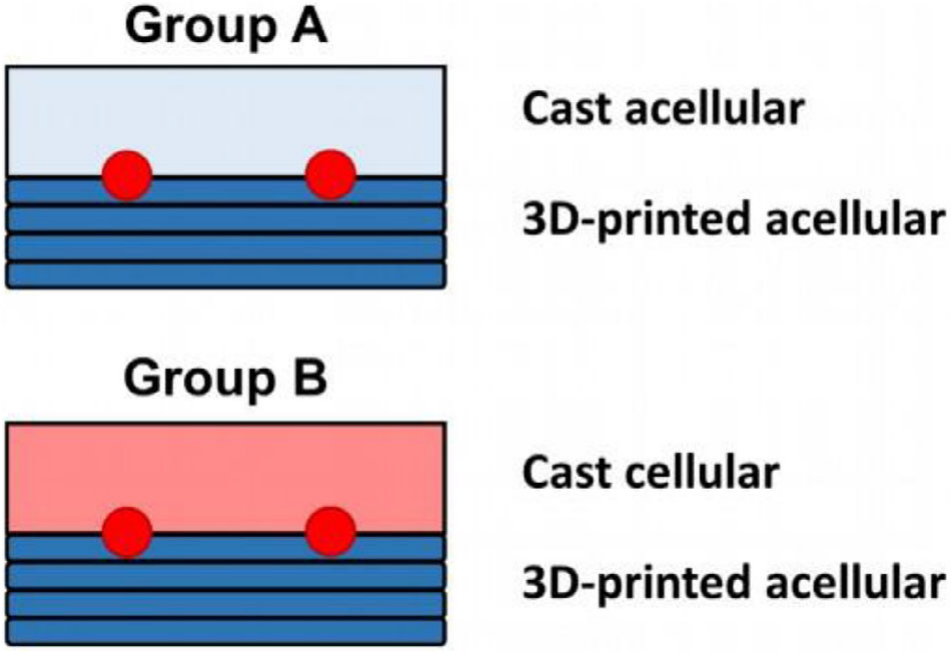
Schematic overview of the experimental setup. The schematic drawing showing a cross‐section of the chamber. Each PTFE chamber is filled with a 3D‐printed eADF4(C16)‐RGD scaffold on the bottom half (dark blue). The AV loop was placed on top in both groups (red). In group A, unprinted bioink of identical composition was added to fill the upper half (light blue). In comparison, the upper half of group‐B‐chambers was filled with unprinted eADF4(C16)‐RGD containing T17b cells (pink).

The animal experiments were approved by the Animal Care Committee of the University of Erlangen and the Government of Mittelfranken (AZ 55.2‐2532‐2‐763) and were carried out according to EU Directive 2010/63/EU. In total, 26 male Lewis rats (Charles River Laboratories, Sulzfeld, Germany) with an initial body weight of 360 to 480 g underwent surgery. Two experimental groups were established: Half of the PTFE chamber was filled with the 3D‐printed eADF4(C16)‐RGD scaffold, in group A the upper half was filled with unprinted bioink of the same composition, without any cellular components, while in group B the second half was supplemented with unprinted bioink containing T17b cells (Figure 11). The implantation‐to‐explantation intervals were set at 2 and 4 weeks with a sample size of n = 4, and at 12 weeks with a sample size of n = 5 for each group.

**FIGURE 12:**
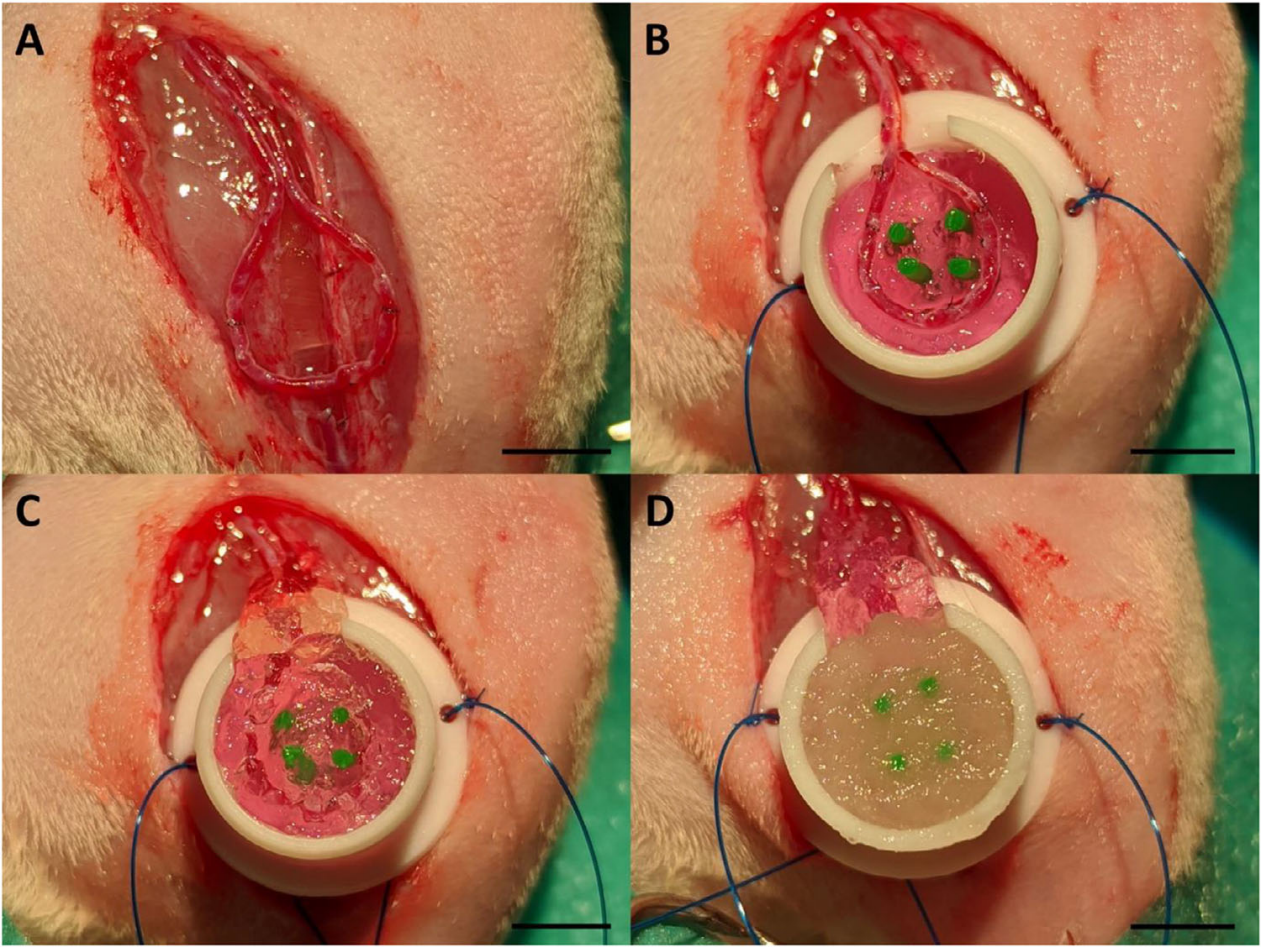
AV loop surgery. After anastomosing the vein graft between the saphenous vessels (A) and placing the AV loop onto the 3D‐printed spider silk hydrogel (B), the PTFE chamber was filled up with cast spider silk without cells (C) or with T17b cells (D). Scale bar = 5 mm.

The AV loop surgery was performed in a sterile operation field. General anesthesia was induced and maintained with isoflurane (GP‐Pharma, Germany). In addition to that, the animals received intravenous perioperative prophylactic antibiotics (enrofloxacin 7.5 mg kg^−1^, Bayer, Germany) and analgesia (butorphanol 1.5 mg kg^−1^, CP‐Pharma, Germany and meloxicam 1 mg kg^−1^, Boehringer Ingelheim Vetmedica GmbH, Germany). After placing the animal on a heating plate and shaving and disinfecting both hind legs, a skin incision was made to expose the saphenous vascular nerve cord. Operating under a surgical microscope (Carl Zeiss, Oberkochen, Germany), the saphenous vein from the right leg was collected and flushed with a heparin solution (500 IU mL^−1^, Ratiopharm, Germany) to prevent blood clotting. This vein graft was then connected to the saphenous artery and vein of the left leg with end‐to‐end anastomosis using interrupted sutures (Ethilon 11–0; Johnson & Johnson, New Brunswick, NJ, USA) forming the AV loop (Figure 12). Subsequently, a PTFE chamber half filled with 3D‐printed spider silk was fixed on the thigh musculature and the AV loop was carefully placed on top. The remaining upper half was filled in with either cast spider silk without cells (group A; Figure 12C), or with spider silk containing differentiated T17b cells per ml (group B; Figure 12D). To prevent the AV loop from kinking or damaging, the entrance of the chamber was padded with excess spider silk (Figure 12C,D). Finally, the chamber was closed with a lid and the wound was closed.

Postoperatively, the animals received enoxaparin sodium (10 mg kg^−1^, Sanofi S.A., France) for 2 days and antibiotics as well as analgesics for 7 days.

During the implantation period, blood samples were taken weekly to check for the presence of fusion protein.

### 5.7 Explantation

After the observation period of 2, 4, and 12 weeks, the PTFE chambers were explanted. Therefore, a median skin incision from sternum to symphysis was made. A peripheral venous catheter (24 G, Introcan Safety‐W, Braun, Melsungen, Germany) was then placed proximally into the descending aorta and fixed with a prepared ligature. The caval vein was incised further distally. To analyze vascularization, an intraarterial perfusion with 100 mL NaCl‐Heparin solution (100 IU mL^−1^, Ratiopharm) followed by 20 mL Microfil MV‐122 solution (Flow Tech Inc., Carver, USA) was carried out. Microfil was prepared as described by the manufacturer, preheated MV compound and MV diluent (1:2) plus 5% curing agent added directly before use. Urine and blood samples were taken during the operation procedure. After overnight storage at 4°C, the constructs were excised, weighed and immersed in Roti‐Histofix 4% (Carl Roth) over night and stored in PBS afterwards.

### 5.8 µCT

In order to demonstrate the patency of the AV loops and to analyze the newly sprouted vessels, high‐resolution µCT scans were performed by a Skyscan 1172 (Skyscan B.V., Leuven, Belgium) with a 11‐megapixel detector and a tungsten tube. The voltage was set to 80 kV and the current to 100 µA and a pixel size of 6.78 µm. Tomographic reconstruction software (NRecon Client and Server 1.6.10.1 with GPU support; Skyscan, Leuven, Belgium) was used to reconstruct the sinograms. Cumulative vessel length was calculated for each construct.

### 5.9 Histological Staining

After passing through µCT, the constructs were embedded in paraffin. They were then cut into 3‐µm‐thick cross‐sections perpendicular to AV loop longitudinal axis using a microtome (Leica Microsystems, Germany). Hematoxylin and eosin (H&E) and alpha‐smooth muscle actin (*α*‐SMA) staining was conducted according to standard protocols to examine the number and structure of the vessels originating from the AV loop [44]. For immunostaining of macrophages, a CD68 staining was used, allowing conclusions about biocompatibility. Therefore, histological tissue sections were incubated with an anti‐CD68 primary antibody (1:300 dilution, BIO‐RAD, Hercules, USA) overnight. Following incubation with post blocking solution, an alkaline phosphatase‐labeled anti‐mouse antibody and Fast Red TR/Naphthol AS (Sigma–Aldrich) were applied for color reaction. Finally, the sections were counterstained with haemalaun.

In order to detect the TNFR2‐Fc‐FLAG‐GpL fusion protein, a FLAG tag staining was per‐formed. After deparaffinization and rehydration, the cross‐sections were boiled in Tris/EDTA buffer (pH 6) for 36 min in order to retrieve epitopes. They were then incubated in blocking solution for 7 min, followed by washing in TRIS buffer. Subsequently, the specimens were incubated for 30 min in a solution of 10% goat serum in PBS. After that, they were incubated overnight at 4°C with a primary antibody (recombinant Anti‐DDDDK tag), diluted 1:750 in antibody diluent. The next day, after washing in TRIS buffer, peroxide block was applied for 15 min. Another washing step was carried out. The sections were then incubated with a secondary antibody (Dako REAL EnVision HRP Rabbit/Mouse (ENV)) for 30 min. For visualization, the sections were stained with DAB substrate for approximately 5 min. This process was terminated by rinsing with demineralized water. Finally, counterstaining was performed by immersing the specimens in a haemalaun solution (Sigma–Aldrich) for 35 s.

Hypoxic conditions were assessed by immunohistochemically detecting HIF‐1*α*. Sections were incubated with a primary anti‐HIF‐1*α* antibody (1:100; clone H1alpha67, NB100‐123; Novus Biologicals, Bio‐Techne GmbH, Wiesbaden‐Nordenstadt, Germany) using citrate buffer (pH 6). Visualization was achieved using a horseradish peroxidase–based DAB detection system (DAKO EnVision, Agilent, USA).

Matrix degradation was evaluated through the immunohistochemical detection of MMP‐3. Deparaffinized histological tissue sections were subjected to antigen retrieval using citrate buffer (pH 6) and incubated with a primary anti‐MMP‐3 antibody (1:50; Sigma–Aldrich). Signal detection was performed using peroxidase–based DAB detection system (DAKO EnVision, Agilent, USA).

For statistical analysis, tissue section images were acquired using either an Olympus IX81 microscope (Olympus, Hamburg, Germany) with cellSens Dimension V1.5 software or a PANNORAMIC 250 Flash scanner (3DHISTECH, Budapest, Hungary) and processed using CaseViewer 2.4 software (3DHISTECH). The number of vessels was counted manually using ImageJ 1.52 p (NIH, Bethesda, Maryland, USA). For measuring construct area and connective tissue area measurements, the interface was marked in GIMP 2.10 (GNU Image Manipulation Program) and calculated in ImageJ.

### 5.10 Protein Measurement

The blood and urine samples taken were tested for luminescence of the TNFR2‐Fc‐FLAG‐GpL fusion protein. For this purpose, the blood samples were first centrifuged (2000 × g, 10 min) and the measurement was carried out in serum. The samples, as well as blank samples, were diluted 1:10 with RPMI‐1640 (Sigma; containing 0.5% FCS and penicillin/streptomycin, 100 U mL^−1^, 0.1 mg mL^−1^; both Sigma–Aldrich) and the relative light units were determined in a luminometer microplate reader (Berthold Technologies, Germany) after the addition of coelenterazine (1.5 µm; Roth). All samples measured in technical triplicates.

### 5.11 Statistical Methods

GraphPad Prism 10.2.0 (GraphPad Software, San Diego, USA) was used for statistical analysis. Initially, the normal distribution of the results was verified. With normal distribution an unpaired Student's t‐test was then used to determine statistically significant differences. For nonparametric test a Mann‐Whitney‐U‐test was used. p‐values ≤ 0.05 were considered significant. Data are expressed as mean ± standard deviation. While the 12‐week AV loops in group A were not fully perfused, the 12‐week constructs of group B had begun to dissolve during removal from the PTFE chamber. Consequently, both 12‐week constructs had to be excluded from histological and µCT analysis.

## Funding

This project was funded by the Deutsche Forschungsgemeinschaft (DFG, German Research Foundation) – Project number 326 998 133 – TRR 225 (subprojects C04, C01). This work was supported by the Interdisciplinary Center for Clinical Research (IZKF) at the University Hospital of Erlangen‐Nuremberg (MD‐Thesis Scholarship Programme) and the Friedrich‐Alexander‐University Erlangen‐Nürnberg.

## Conflicts of Interest

The authors declare no conflicts of interest.

## Supporting information




**Supporting File**: adhm70896‐sup‐0001‐SuppMat.docx.

## Data Availability

The data that support the findings of this study are available from the corresponding author upon reasonable request.
