# Supplementary material for: Biofabrication of Endothelialized, Intrinsically Vascularized 3D‐Printed Recombinant Spider Silk Scaffolds
Source: Adv Healthc Mater. 2026 Feb 8;15(16):e04883. doi: 10.1002/adhm.202504883 (PMC13107924; doi:10.1002/adhm.202504883)
Supplement: Supplementary file 1 — Supporting File: adhm70896‐sup‐0001‐SuppMat.docx. [file ADHM-15-0-s001.docx]

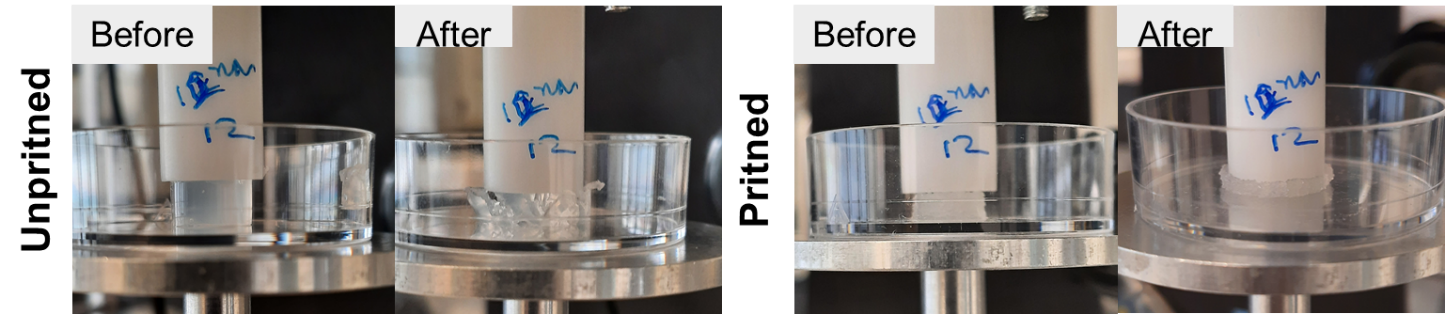


**Supplementary figure 1: Appearance of hydrogels before and after the compression tests** showing the difference in material failure. Unprinted hydrogel samples underwent drastic fracturing compared to the flow-deformation of printed constructs.
